# Supplementary material for: Causal Relationship between Aging and Anorexia Nervosa: A White-Matter-Microstructure-Mediated Mendelian Randomization Analysis
Source: Biomedicines. 2024 Aug 16;12(8):1874. doi: 10.3390/biomedicines12081874 (PMC11351342; doi:10.3390/biomedicines12081874)
Supplement: Supplementary file 1 [file biomedicines-12-01874-s001.zip › Supplementary Figure/Figure S4/Singe SNP plot of MR analysis for TL to ubm-b-1634.pdf]

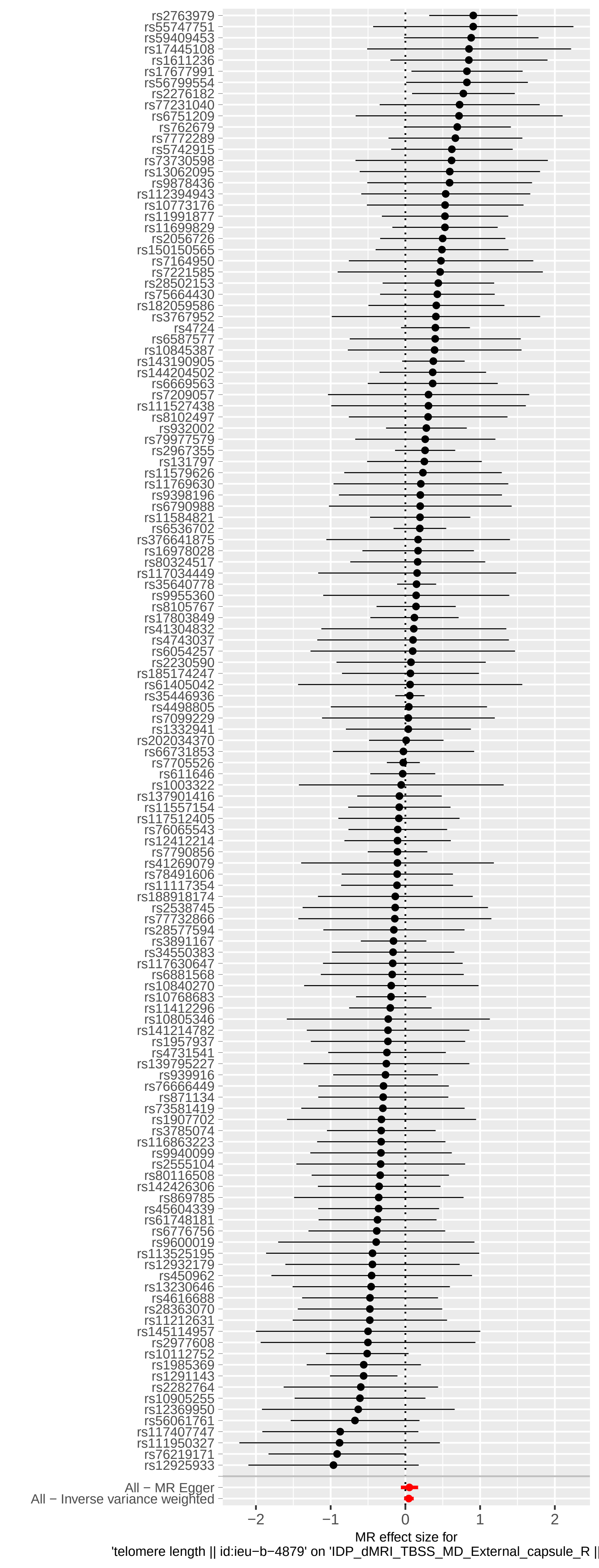

rs2763979
rs55747751
rs59409453
rs17445108
rs1611236
rs17677991
rs56799554
rs2276182
rs77231040
rs6751209
rs762679
rs7772289
rs5742915
rs73730598
rs13062095
rs9878436
rs112394943
rs10773176
rs11991877
rs11699829
rs2056726
rs150150565
rs7164950
rs7221585
rs28502153
rs75664430
rs182059586
rs3767952
rs4724
rs6587577
rs10845387
rs143190905
rs144204502
rs6669563
rs7209057
rs111527438
rs8102497
rs932002
rs79977579
rs2967355
rs131797
rs11579626
rs11769630
rs9398196
rs6790988
rs11584821
rs6536702
rs376641875
rs16978028
rs80324517
rs117034449
rs35640778
rs9955360
rs8105767
rs17803849
rs41304832
rs4743037
rs6054257
rs2230590
rs185174247
rs61405042
rs35446936
rs4498805
rs7099229
rs1332941
rs202034370
rs66731853
rs7705526
rs611646
rs1003322
rs137901416
rs11557154
rs117512405
rs76065543
rs12412214
rs7790856
rs41269079
rs78491606
rs11117354
rs188918174
rs2538745
rs77732866
rs28577594
rs3891167
rs34550383
rs117630647
rs6881568
rs10840270
rs10768683
rs11412296
rs10805346
rs141214782
rs1957937
rs4731541
rs139795227
rs939916
rs76666449
rs871134
rs73581419
rs1907702
rs3785074
rs116863223
rs9940099
rs2555104
rs80116508
rs142426306
rs869785
rs45604339
rs61748181
rs6776756
rs9600019
rs113525195
rs12932179
rs450962
rs13230646
rs4616688
rs28363070
rs11212631
rs145114957
rs2977608
rs10112752
rs1985369
rs1291143
rs2282764
rs10905255
rs12369950
rs56061761
rs117407747
rs111950327
rs76219171
rs12925933
All − MR Egger
All − Inverse variance weighted
−2
−1
0
1
2
MR effect size for
'telomere length || id:ieu−b−4879' on 'IDP_dMRI_TBSS_MD_External_capsule_R ||
